# Role of clinical research coordinators in promoting clinical trials of drugs for surgical patients

**DOI:** 10.1186/1755-7682-1-26

**Published:** 2008-12-10

**Authors:** Hiroaki Yanagawa, Akiyo Akaishi, Toshiko Miyamoto, Shigemi Takai, Rika Nakanishi, Minoru Irahara

**Affiliations:** 1 Clinical Trial Center for Developmental Therapeutics, Tokushima University Hospital, Tokushima, Japan

## Abstract

**Background:**

Clinical trials play a central role in the establishment of clinical evidence, and the important role of clinical research coordinators (CRCs) in various processes of clinical trials is now widely recognized. In Japan, many CRCs work under the discretion of their hospital and support clinical trials in various areas. Modification of CRC activity pursuant to the types of clinical trials may make roles of the CRC more effective and meaningful. In the present study, we examine the dedicated role of the CRC considering the specialty of a registration trial of a drug for surgical patients used during the operation period.

**Methods:**

In 2006, we had a chance to support a registration trial of a drug for surgical patients used during the operation period. Regarding the mental and emotional status of possible participants in the present registration trial, we collected data from the perspective of CRCs by focus group interviews involving four CRCs working under the discretion of Tokushima University Hospital. The four CRCs were all nurses and had 7, 4.5, 1, or 0.5 years experience as CRCs, respectively.

**Results:**

In contrast to clinical trials of drugs for chronic diseases, these often anxious patients must decide whether or not to enter the trial simultaneously with the decision to undergo surgery itself, and all in a relatively limited time after receiving explanation of the trial. Therefore, special attention should be paid to the mental and emotional status of possible participants. Additionally, the cooperation of the relatively large surgical and nursing staff becomes important. In such situations, the following contributions of CRCs were considered to be useful for the harmonious procedure of clinical trials: 1) providing a precise explanation of the trial to the participant and key persons, 2) understanding the needs of the investigators and appropriately assigning themselves roles, and 3) communicating between the investigators and surgical and nursing staff.

**Conclusion:**

Further study is warranted to evaluate the benefit of the intervention provided by dedicated CRCs in running high quality clinical trials involving surgical patients.

## Background

Clinical trials play a central role in the establishment of clinical evidence, including proper evaluation of effect and safety profiles of drugs in the development process. In the implementation of clinical trials, the importance of the contribution of clinical research coordinators (CRCs) is now widely recognized. CRCs are responsible for screening and recruiting participants, ensuring informed consent, collecting and recording data and participant follow-up. In addition, CRCs have a critical role in protecting human subjects in clinical trials and the involvement of CRCs results in efficient progress and improved quality of clinical trials [1-3]. In western countries, CRCs work within clinical departments, such as hospital wards, and are responsible for the management of research undertaken in that setting. As such, they specialize in a field of clinical research, such as oncology or cardiology [4]. In Japan, CRCs work under the discretion of the site management organization (SMO) and work on each trial on a basis similar to western countries. However, Japan differs from western countries in that many CRCs work under the discretion of their hospital, and, in general, support clinical trials in various areas. Modification of CRC activity pursuant to the types of clinical trials may make roles of the CRC more effective and meaningful. Furthermore, compared to CRCs in a specialized area, "general" CRCs have less of a chance of being in contact with clinical trials in each area, and sharing experiences and participating in support activities in various areas among CRCs may be necessary to overcome this deficit.

At Tokushima University Hospital, an academic hospital in the Shikoku district of Japan, the Clinical Trial Center for Developmental Therapeutics (CTCDT) was set up in 1999 to support clinical trials [5,6]. CRCs in our hospital work under the discretion of the CTCDT to support all registration trials, regardless of the investigators' department, area, or drug type. In 2006, we had the opportunity to support a registration trial for a drug administered to patients during surgery. In contrast to clinical trials of drugs for chronic diseases, we feel that special attention should be paid to the mental and emotional status of possible surgical participants. It is useful to share information regarding the specialty of clinical trials of drugs for surgical patients among CRCs in various hospitals, and we examined the role of the CRC in that type of clinical trial in the present study.

## Methods

Regarding the mental and emotional status of possible participants in the present registration trial, we collected data from the perspective of the CRC by focus group interview, since it was expected to be superior to individual interviews [7,8]. In Tokushima University Hospital, all CRCs supporting registration trials under the CTCDT were included in and contributed as investigators to the study. They were all nurses and have 7 years (CRC1), 4.5 years (CRC2), 1 year (CRC3), and 0.5 year (CRC4) experience as CRCs respectively. Several group interviews were facilitated by the vice-director of the CTCDT in 2006. In order to avoid the observer dependency and to respect the group dynamics, the observer only mentioned the following topics. The first topic was: "what is the difference in the situation of possible participants between those with chronic diseases and surgical patients?" After collecting general remarks, group members were encouraged to express their views on how to best protect participants in these settings. Another topic was the difference in involvement and contribution of hospital staff to the efficient progress and improved quality of clinical trials between chronic disease patients and surgical patients. After discussion, possible measures considering each difference were suggested distinguishing those specific to Tokushima University Hospital and those that could be generalized. Data are presented to include illustrative quotations and examples of dialogue between participants.

## Results

### 1. Situational differences between candidates who are going to undergo surgery and candidates for clinical trials involving chronic diseases

All CRCs agreed that the role of the CRC is very different among the types of clinical trials and the role in registration trials for surgical patients is different from that in trials for chronic disease patients treated in an out-patient clinic.

Some CRCs had experienced as nurses that patients are anxious about the outcome of the operation itself in the preoperative period (CRC1, CRC4). Candidates have to process information concerning the registration trial and must decide whether or not to participate while coping with anxiety about the operation itself. Moreover, they have to decide in a relatively short period of time when compared to registration trials involving chronic diseases. In general, the explanation for the operation and related matters is done by ward-based clinical nurses, and CRCs explain matters concerning clinical trials. Sometimes they are done together (e.g., on the same day) and that can confuse patients.

### 2. Differences regarding the involvement and contribution of hospital staff to the efficient progress and improved quality of clinical trials between chronic disease patients and surgical patients

The main difference is that more departments are involved in clinical trials involving surgical patients than in clinical trials involving chronic disease patients. In the registration trial that we supported, the principal investigator worked under the discretion of the Surgery Center of Tokushima University Hospital, and the need to communicate closely with the doctors of the surgery departments was emphasized by all CRCs. As for the nursing staff, those of the outpatient clinic are mainly involved in clinical trials for outpatients. In contrast, nursing staff members from two or more areas are involved in clinical trials for surgical patients. All CRCs agreed that close communication between clinical nurses of the ward and surgery center was important. CRC1 suggested that insufficient consultation about the trial to the ward staff sometimes resulted in poor compliance, and in the ward staff relying exclusively upon the CRC to deal with aspects of clinical trials [laughter and agreement in the group].

As for collecting and recording data, the anesthesia record was used separately in surgery patients in addition to the common medical record in Tokushima University Hospital. All CRCs agreed that in clinical trials involving surgical patients (rather than chronic patients), the contribution of more professionals in more departments is necessary to follow protocol and to collect sufficient data. CRCs have an important role to play in integrating them.

### 3. Measures considering various differences and important points

#### 1) Measures for participants

CRCs introduce themselves to participants for the registration trial and inform patients that the explanation of the registration trial will be performed by CRCs, while clinical nurses will separately explain matters relating to the treatment (operation), to avoid confusion. CRCs provide time for participants to consult with the key person concerning the registration trial and to encourage the participant to ask the key person to explain the opportunity. CRCs visit participants at a time that will interfere with neither preoperative examinations nor explanation of matters pertaining to the treatment (operation) by clinical nurses.

#### 2) Measures for staff of the surgery center

The operation period is the main focus of the registration trial; cooperation of all surgery center professionals is necessary to ensure protocols are followed and participants are monitored properly. Firstly, CRCs must become acquainted with the various systems of the surgery center (with which CRCs are not always familiar). CRCs explain the registration trial repeatedly to the surgical team, with special reference to the difference between standard clinical practice and issues particular to the registration trial. The schedule of the operation day should be clearly shown on a special sheet and should be issued to all professionals of the surgery center and the CRCs should provide information concerning predictable events that may occur after the use of an investigational drug.

#### 3) Measures for surgeons and surgery ward staff

Since the registration trial that we supported regarded a drug for surgical patients used by the anesthesiologist during the operation, surgeons and surgery ward staff had no direct contact with the investigational drug. It is the role of the CRCs to explain the framework of the trial to the surgery department staff. When CRCs obtain informed consent from patients, they convey this to surgeons and surgery ward staff. Since participants will be cared for and monitored by ward-based clinical nurses after short-term follow-up by clinical nurses of the surgery center, CRCs provide information to ward staff concerning predictable events that may occur after the use of the investigational drug.

## Discussion

It is now widely accepted that CRCs play important roles in ensuring the quality of clinical trials while lessening the workload of physicians. The main task of the CRC, irrespective of their background, is patient monitoring. Nevertheless, nurses can perform more activities than CRCs without a clinical background. It is important to consider activities of CRCs in light of their backgrounds and experiences in clinical trials. For example, CRCs of critical care units must be skilled in the process of obtaining informed consent with unconscious and/or intubated patients, as well as maintaining a setting where patients may enter complex research protocols 24 hours a day, 7 days a week [9]. The role of CRCs in the neonatal intensive care unit (NICU) has been reported by Thompson et al. [10]. In the present paper, we examine the differences between the roles of CRCs in clinical trials of drugs used for surgical patients versus those in clinical trials for outpatients.

In Japan, the establishment of both national and regional networks for clinical trials among medical institutions is emphasized in the plan for promoting registration trials by the Ministry of Health, Labor and Welfare, and by the Ministry of Culture and Science of Japan. This is now spreading as a means of promoting clinical trials. In 2004, in order to spread information of the supporting system of the CTCDT in the regional area, Tokushima University Hospital, in collaboration with the Tokushima Medical Association, set up the Tokushima Network for Clinical Trials (TNCT) which comprises regional medical institutions [11]. The TNCT's mission is to gain a clearer understanding of the methodology in support of clinical trials in various areas and situations and to promote clinical trials in this rural area of Japan. Since most medical institutions registered with the TNCT have no experience with registration trials like those on which the present study focuses, we conducted the present study with discussion among four CRCs working under the discretion of Tokushima University Hospital.

The present analysis revealed the importance of attending to the mental and emotional status of possible participants since these patients must decide under stressful circumstances whether or not to enter the trial while simultaneously deciding whether or not to undergo surgery itself, and all in a relatively limited time after receiving explanation of the trial. To provide time and encourage surgical patients to consult the key person concerning the registration trial can be one possible measure considering the mental and emotional status of them. This study also illustrates the importance of cooperation of the relatively large surgical and nursing staff throughout the trial. In such situations, the following contributions of CRCs were considered to be useful for the harmonious procedure of clinical trials: 1) providing a precise explanation of the trial to the participant and key persons and 2) facilitating communication between the investigators and the surgical and nursing staff.

From the perspective of the CRC, ward-based clinical nurses work as a nursing team, and CRCs must have competence for their research role and must adapt to working alone and working with a variety of clinical professionals. CRCs often feel insecure and feel that they are perceived as a minority group, and that their complaints cannot be accepted by colleagues who lack understanding and insight into the research process [12]. Feelings of isolation and tension throughout the clinical trials exist even after CRCs have gained skills and confidence in their roles [13]. Mueller and Mamo [14] identified three major themes as having both benefits and drawbacks in the position of CRC: work autonomy, relationship with trial patients and with the investigator, and clinical or technical skills and knowledge. CRCs have more time to share with study participants than other staff, and the additional contributions by CRCs may make trial participants more familiar with their disease and treatment, and acknowledgement of that contribution may make CRCs more confident in their roles. These possibilities should be examined in future studies.

The TNCT conducts seminars regularly for practical training in the methodology of registration trials for physicians and medical specialists (including CRCs). Using these seminars, we are now working to share the findings we obtained in the present study among CRCs working under the discretion of medical institutions affiliated with the TNCT. To evaluate and generalize the present findings, it is recommended that the ideal role of CRCs is examined. This can be extended to CRCs working with surgical patients in many groups in various medical institutions in the region and at large.

## Competing interests

The authors declare that they have no competing interests.

## Authors' contributions

HY conceived of the study, collected data and analyzed them, and drafted the manuscript. AA, TM, ST and RN participated in the design of the study and contributed to the interview. MI participated in the design of the study and helped to draft the manuscript. All authors read and approved the final manuscript.

## References

[B1] Isaacman DJ, Reynolds EA (1996). Effect of a research nurse on patient enrolment in a clinical study. Pediat Emerg Care.

[B2] Kenkre J, Chatfield D (2004). Study site co-ordinator/clinical research nurse. Clin Res Focus.

[B3] Davis AM, Hull SC, Grady C, Wilfond BS, Henderson GE (2002). The invisible hand in clinical research: The study coordinator's critical role in human subjects protection. J Law Med Ethics.

[B4] Rickard CM, Roberts BL, Foote J, McGrail MR (2007). Job satisfaction and importance for intensive care unit research coordinators: results from binational survey. J Clin Nurs.

[B5] Yanagawa H, Irahara M (2003). Supporting system for promotion of clinical trials at Tokushima University Hospital. Shikoku Acta Medica.

[B6] Yanagawa H, Nishiya M, Miyamoto T, Shikishima M, Imura M, Nakanishi R, Ariuchi N, Akaishi A, Takai S, Abe S, Kisyuku M, Kageyama C, Sato C, Yamagami M, Urakawa N, Sone S, Irahara M (2006). Clinical trials for drug approval: a pilot study of the view of doctors at Tokushima University Hospital. J Med Invest.

[B7] Basch CE (1987). Focus group interview: an under-utilized research technique for improving theory and practice in health education. Health Educ Q.

[B8] Powell RA (1996). Methodology matters, focus groups. Int J Qual Health Care.

[B9] Jones CM, Mitchell-Inwang C (2002). New roles for nurses in critical care: the research nurse practitioner. Care Crit Ill.

[B10] Thompson A, Pickler RH, Reyna B (2005). Clinical coordination of research. Appl Nurs Res.

[B11] Yanagawa H, Irahara M, Kawashima S, Kagawa S, the Tokushima Network for Clinical Trials (2008). The views of doctors on registration trials in a Japanese rural area: a survey of medical institutions registered to the Tokushima Network for Clinical Trials. J Int Med Res.

[B12] Raja-Jones H (2002). Role boundaries – research nurse or clinical nurse specialist? A literature review. J Clin Nurs.

[B13] Spilsbury K, Petherick E, Cullum N, Nelson A, Nixon J, Mason S (2007). The role and potential contribution of clinical research nurses to clinical trials. J Clin Nurs.

[B14] Mueller MR, Mamo L (2002). The nurse clinical trial coordinator: benefits and drawbacks of the role. Res Theory Nurs Pract.

